# A Pilot Study to Evaluate the Potential of Melatonin Implants to Control Cyclicity in the Bitch

**DOI:** 10.3390/ani13081316

**Published:** 2023-04-11

**Authors:** Eva Axnér

**Affiliations:** Department of Clinical Sciences, Division of Reproduction, Faculty of Veterinary Medicine and Animal Science, Swedish University of Agricultural Sciences, P.O. Box 7054, SE-75007 Uppsala, Sweden; eva.axner@slu.se

**Keywords:** reproduction, oestrus, canine, dog, female, melatonin

## Abstract

**Simple Summary:**

Current methods to control the reproductive cycle in the bitch are associated with problematic side effects. Melatonin plays a role in the regulation of reproduction in several species. It has been used to control the reproductive cycle in species with seasonal reproduction. The ancestor of the dog, the grey wolf, has strict seasonal reproduction with breeding in early spring. In contrast to the wolf, the domestic dog is a non-seasonal breeder. It is still not known whether melatonin is involved in control of cyclicity in either the wolf or the domestic dog. The aim of this study was, therefore, to evaluate whether a long-acting melatonin implant would be a safe alternative for short-term postponement of heat in the bitch. Five beagle bitches were treated with melatonin implants while another four bitches were untreated controls. There was no effect of treatment with melatonin implants on the cyclicity in this study. Implants with 18 mg are, therefore, not likely to be a useful method to control cyclicity in the bitch, in contrast to some other species. It is still not known whether melatonin is involved in regulation of the oestrus cycle in the domestic dog.

**Abstract:**

In short-day breeders such as the sheep, melatonin stimulates oestrus activity; in contrast, a high serum concentration of melatonin inhibits oestrus in long-day breeders such as the cat. Therefore, implants with melatonin have been used to suppress or induce oestrus depending on the species. The aim of this pilot study was to evaluate if melatonin could be an alternative to control the reproductive cycle in the bitch. Nine beagle bitches were observed for three oestrus cycles. Five beagle bitches were treated with 18 mg melatonin implants on average 27 days before the next expected oestrus based on the previous interoestrus interval. Four bitches served as untreated controls. Blood samples for evaluation of serum melatonin were collected at the time of assignment to treatment or control group and 1–4 weeks thereafter. Clinical signs and vaginal smears were used to follow the cycle. Melatonin varied significantly with bitch (*p* < 0.05) but not with treatment. Treatment did not affect the interoestrus interval (*p* > 0.05). In conclusion, treatment with 18 mg melatonin implants approximately one month before expected oestrus is not likely to be a useful method to control cyclicity in the bitch. It is still not known if melatonin is involved in regulation of the oestrus cycle in the domestic dog.

## 1. Introduction

Current methods to control the oestrus cycle in the bitch are associated with problematic side effects. Ovariohysterectomy (OHE, spaying) offers a permanent solution. However, for bitches intended for future breeding, OHE is obviously not an alternative. Although OHE is associated with advantages, it is also associated with some side effects that vary with breed and individual [1].

Gestagens can be administered for short- or long-term control of oestrus. Because of the risk of serious side effects [2], use of gestagens is not very popular; although, in the absence of other alternatives, it may sometimes be the best alternative for postponing oestrus.

Deslorelin (Suprelorin implant 4.7 mg and 9.4 mg, Virbac, Carros, France) is a depot long-acting GnRH agonist. Suprelorin is associated with an initial stimulatory effect. Initially, the long-acting deslorelin implants stimulate the release of FSH and LH from the hypothalamus before downregulation, caused by desensitization of GnRH receptors, occurs. The initial “flare-up” effect may induce oestrus and has been associated with a risk of inducing uterine pathologies [3]. Persistent heat has also been reported as a side effect in bitches [3]. Because of the initial stimulatory effect and the subsequent long-term downregulation of ovarian function, depot GnRH agonists are not practical for short-term postponment of oestrus in the bitch.

An alternative to controlling the oestrus cycle by postponing oestrus is to instead induce oestrus earlier than the natural oestrus would occur. Oestrus can be induced off-label by administering cabergoline (Galastop^®^ vet, Ceva Santé Animale, Libourne, France). The success rate is around 80% [4]. Because of the lack of effect in some bitches and the large variability in time until effect, the use of cabergoline to control the oestrus cycle is sometimes not practical. In addition, the treatment can be expensive in large bitches, as the duration of treatment until effect sometimes can take up to more than 40 days.

Melatonin is secreted by the pineal gland and is regulated by the amount of light. In short-day breeders such as the sheep, high serum concentration of melatonin stimulates oestrus activity, whereas a high serum concentration inhibits oestrus in long-day breeders such as the cat [5,6,7,8,9]. Melatonin may interfere with oestrogen receptors and may also alter the secretion of FSH and LH. Human reproductive organs have receptors for melatonin [5]. A long-acting implant (Regulin^®^ Ceva Animal Health Pty Ltd Glenorie, Australia), releasing melatonin for 100–120 days, is licensed in some countries to induce oestrus in sheep [10].

In the domestic cat, melatonin implants (18 mg/cat s.c), licensed for use in sheep, resulted in a significantly prolonged interoestrous interval compared with untreated controls (63.8 days for treatment group vs. 19.2 days for control group). Similar results were achieved with oral melatonin (4 mg/cat/day) [7]. Gimenez et al. [6], using the same type of melatonin implants, achieved suppression of oestrus for 2–4 months in queens without any side effects. Male cats are also affected by season [11], and melatonin implants (18 mg) significantly and reversibly decrease sperm quality [12], in contrast to rams, where it is claimed to increase sperm production [10,13]. No side effects have been reported after treatment with melatonin implants in the domestic cat [7,12]. Melatonin is considered a safe drug with a very low risk of serious side effects [14].

The ancestor and close relative of the dog, the grey wolf (*Canis lupus*), has strict seasonal reproduction with breeding in early spring. Wolves breed earlier at lower latitudes, suggesting an effect of photoperiod [15]. In contrast to the wolf, the domestic dog is considered a non-seasonal breeder with an average of two cycles per year (1–4), with no evidence of a seasonal pattern [16]. However, some traces of seasonality in some breeds of domestic dogs have been suggested, with more and larger litters born in the spring [17]. In contrast to other breeds of domestic dogs, the Basenji is known to be a seasonal autumn breeder. This is in contrast to the wolf that breeds during early spring. Oestrus in the Basenji is induced by decreasing day length and thus controlled by photoperiod [18]. It is generally considered, however, that during the process of domestication, most breeds of dogs lost photoperiodic control of the cycle [19].

There are some data suggesting a possible effect of melatonin on canine seasonality. Dunlap et al. [20] found that serum melatonin in the dog was higher in the winter than in the summer [20]. Oral administration of melatonin, in doses of 1.0 to 1.3 mg/kg of body weight every 12 h for 28 days, was associated with a significant decrease in serum oestradiol in five bitches. Four of the bitches were in dioestrus, and the other two were likely in anoestrus at the start of treatment in their study. The effect of melatonin on the oestrous cycle in the bitch was unfortunately not reported [21]. In addition, mean serum oestradiol concentrations did not seem to be lower in the treatment group compared to three untreated control bitches, which is why the effect on reproductive hormones is considered unclear. In intact male dogs, oral treatment with melatonin significantly decreased serum estradiol levels and increased serotonin concentrations [22]. There is thus a paucity of information on the potential effects of melatonin on dog reproduction. The decreased serum oestradiol concentrations after administration of melatonin indicates, however, that long-acting melatonin implants might have the potential to postpone oestrus if administered in late anoestrus [21]. On the other hand, a stimulating effect by decreasing day length in the Basenji breed would indicate a stimulating effect of melatonin similar to that seen in sheep [18]. It would thus be of great interest to further elucidate the possible effects of melatonin on dog reproduction and its possible potential for postponing oestrus.

Administration approximately one month before the expected oestrus would be practical, as a bitch that comes into oestrus before that period is likely to be out of oestrus before the start of a hunting period or a planned vacation. It would be close enough to the time of the desired effect to be effective.

The aim of this pilot study was, therefore, to evaluate whether long-acting melatonin implants could be of interest as a safe alternative for short-term postponement of oestrus in the bitch.

## 2. Materials and Methods

### 2.1. Animals

Eleven bitches were enrolled at the start of the study. One bitch was excluded because of previous irregular heats and one because she was affected by myositis after the start of the study. Nine beagle bitches, aged 6 to 9 years, were thus available to complete the study. Included bitches had cycled regularly according to records kept by the staff. Ultrasound of reproductive organs was not performed routinely before inclusion. One of the control bitches had given birth to 7 puppies by caesarean section one year before inclusion. Another control bitch had given birth to 5 puppies by caesarean section 4 years before inclusion. She was mated again at the first oestrus included in the study but did not become pregnant. Ultrasound one month after mating revealed a normal uterus and ovaries. She was included in the study based on normal cyclicity and normal reproductive organs. The bitches were part of the beagle colony at the university, were kept in their normal environment, and participated in their normal activities during the study period. This included participating in teaching of veterinary students but not in activities that were considered to have a potential to interfere with the study. The bitches were group housed and had access to outdoor enclosures during the daytime, in addition to being walked on a leash at least three times a week. They were fed twice a day with Hill’s Science Plan Canine Adult Medium. The rooms where the bitches were kept had large windows, allowing for seasonal fluctuations in the hours of day light, in addition to artificial light in the rooms between 07:00 and 21:00.

### 2.2. Study Design

The bitches were observed in two oestrus periods (oestrus 1 and oestrus 2) before treatment to record their interoestrus interval before administration of melatonin. When clinical signs and vaginal smears indicated pro-oestrus, vaginal cytology samples were collected every second to third day until the vaginal smear was typical for metoestrus. Bitches were randomly assigned to one of two treatment groups by rolling a die. The bitches entered the treatment or the control group 12–33 days (mean 28 days) before the next expected oestrus (oestrus 3), based on the previous interoestrus interval, with one exception. The exception was a bitch that started to show signs of heat before she was assigned to the control group. Long-acting melatonin (Regulin 18 mg implant, CEVA Animal Health AB) implants were placed s.c. in 5 of the bitches (treatment). The other group of 4 bitches were sham implanted (empty needle, controls). 

### 2.3. Blood Samples

Blood samples were collected from the cephalic vein after the start of heat, every second or third day during heat until the day vaginal cytology indicated metoestrus, at the time of assignment to treatment or control group, and at a time-point post-treatment or post-control 1–4 weeks later. Venous blood was collected, and serum was separated by centrifugation. Serum was kept frozen in duplicates at −80 °C until analysis. All blood samples were collected during daytime, between 8:00 and 17:00. Some “extra” samples were also collected outside the above schedule. The reason for this was to have extra samples to test the melatonin kit before the scheduled samples were used. Not all the collected blood samples were suitable for evaluation of melatonin because of a too small volume of serum and/or values outside the range and too little serum left to reanalyse the sample after dilution.

### 2.4. Evaluation of Serum Melatonin and Progesterone Concentrations

Serum concentrations of progesterone were analyzed by chemiluminescence (Immulite, Siemens Healthcare Diagnostics, Erlangen, Germany), in blood samples collected the first day that the bitch was in metoestrus (after oestrus 2 and 3), as indicated by vaginal cytology, to confirm that the bitch had ovulated. 

Melatonin was analysed with a commercial Canine Melatonin ELISA Kit (83-MBS738843 Canine Melatonin Elisa kit, MyBioSource, Inc., San Diego, USA). According to the manufacturer, the intra-assay and inter-assay coefficients of variation are <10% and the sensitivity 1 pg/mL. Samples were run in duplicate and samples with high values were reanalysed after dilution. Means were calculated and used for statistical evaluations.

### 2.5. Statistics

A sample-size calculation was performed from estimated data on interoestrus intervals. To detect a postponement with at least 30 days, a power of 0.8, an alpha of 0.05, and an estimated standard deviation of the difference of 15 days, a minimum of 4 bitches was required for a paired test. The interoestrus intervals between non-treated cycles and treated cycles were compared with a mixed-effect ANOVA. Days were log transformed to achieve a normal distribution. Bitch was included as a random factor and treatment (untreated or melatonin treatment) and interval (between oestrus 1 and 2 or between 2 and 3) as fixed factors. Serum concentration of melatonin was evaluated with mixed-effect ANOVA, with bitch as random factor and sample (at treatment/control or post-treatment/post-control) as a fixed factor. The effect of season was also evaluated from all melatonin values (also samples not included in the above), with bitch as a random factor and season as a fixed factor. Season was classified as winter (December, January, February), spring (March, April, May), summer (June, July, August), or autumn (September, October, November). Melatonin values were log transformed to achieve a normal distribution. Progesterone concentrations from samples collected the first day cytological metoestrus was detected in heat 2 and 3 were compared with a mixed-effect ANOVA, with bitch as random factor and treatment group and cycle (metoestrus 2 or 3) as fixed factors. Distribution of the residuals was checked with the Ryan-Joiner test. Results are reported as means ± SD, unless stated otherwise. All calculations were made with Minitab^®^ 19 (Minitab Inc., State College, PA, USA). 

## 3. Results

### 3.1. Interoestrus Intervals

Intervals between oestrus periods are shown in Table 1. The median intervals between the first and second heat were 204.5 (IQR 61.3) and 240.0 (IQR 58.0) days for control and treatment groups, respectively. Corresponding intervals between second and third heat (after treatment) were 207.5 (IQR 6.75) and 232.0 (IQR 50.5) days. The interoestrus intervals differed significantly between bitches (*p* = 0.047) but were not significantly affected by treatment with melatonin (*p* = 0.72) and did not differ between the first and the second intervals (*p* = 0.51). With one exception, individual interoestrus periods were relatively regular, with a difference of 4–14 days between the first and second intervals. One of the control bitches came into oestrus before she was sham implanted. Her second interoestrus interval was 58 days shorter than her first, thus her third oestrus occurred before she was scheduled to enter the control group. 

### 3.2. Serum Concentration of Melatonin

**Figure 1:**
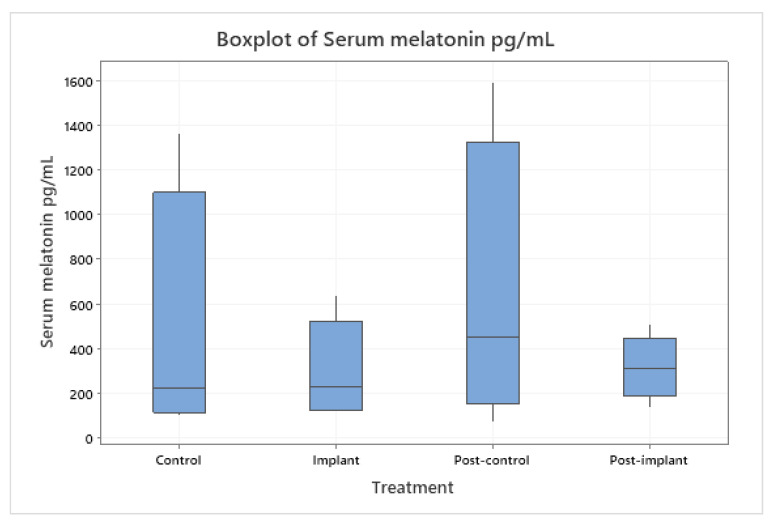
Boxplot of serum melatonin in the control (n = 4) and the treatment (n = 5) groups. Control/Implant refers to samples collected on the day of implant or sham implant. Post-control/Post-implant refers to blood samples collected at one time-point 8–30 days after the Control/Implant samples. There were no significant differences between the groups (*p* > 0.05).

**Figure 2:**
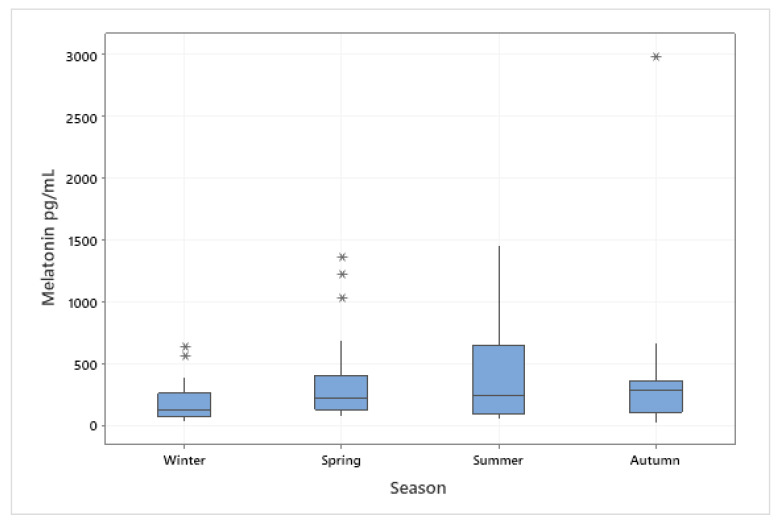
Boxplot of serum melatonin in different seasons. There were n = 9 bitches and 121 observations. There were no significant differences between seasons (*p* > 0.05). * represent outliers.

The measured intra-assay coefficients of variation were 12.3–23.4% (mean 18.4%). There was a large variation in serum concentrations of melatonin with a range between 14.2 and 3219 pg/mL (mean 288.7 ± 336.9) in all samples. There was thus no significant increase in serum melatonin after implantation (Table 2). Two bitches were already in heat at the time of their post-treatment samples. The other seven were in anoestrus, as judged by clinical signs and vaginal cytology. Serum melatonin varied significantly with bitch (*p* = 0.044) but not with treatment (*p* = 0.58, Figure 1). Additionally in the evaluation of the effect of season, serum melatonin concentrations varied significantly with bitch (*p* = 0.043) but not with season (*p* = 0.74, Figure 2).

### 3.3. Serum Concentration of Progesterone

Serum progesterone at the first-day vaginal cytology indicated metoestrus varied between 30.1 and 78.7 nmol/L (median 54.0 nmol/L, SD 18.2), with one exception mentioned below. It did not differ significantly between control and treatment group or between cycles. One of the bitches in the treatment group (bitch number 5 in Table 1) had a low progesterone value at the beginning of her second metoestrus (5.8 nmol/L). A repeated sample one month later was also lower than expected in mid-luteal phase (12.8 nmol/L) but above basal values. The progesterone concentration at her next cycle (after treatment) was 43.6 nmol/L, suggesting that she had a normal ovulation. The statistics for progesterone values were recalculated without this low value, with the same results (i.e., *p* > 0.05 for treatment group and for cycle).

## 4. Discussion

Treatment with melatonin implants did not appear to have an effect on the oestrus cycle based on the results from this study. There was no significant difference in interoestrus intervals between control and treatment groups or between pre- and post-treatment intervals within the treatment group. There was no effect of season on serum concentration of melatonin.

It is unclear from the scarce previous publications on canids on what effect, if any, melatonin could be expected to have on the oestrus cycle of the domestic dog. Although the grey wolf (*Canis lupus*) is known to be a seasonal breeder, neither pinealectomy nor superior cervical ganglioectomy alters puberty or cyclity [23,24]. Oral melatonin decreased prolactin levels in wolves in May to June, when they were at their highest, but not in October to December. Oral melatonin did not affect other reproductive events [24]. Long-acting melatonin implants resulted in an immediate reduction of serum prolactin in the male silver fox and also advanced the breeding season [25]. In contrast to results in the wolf and silver fox, melatonin had no effect on serum prolactin levels in five females dogs kept in a cycle of 12 h of light and 12 h of darkness [21]. Oral melatonin administration was demonstrated to result in increased serum serotonin concentrations in the domestic dog [22], which is expected to cause an increase in prolactin concentrations rather than the decrease observed in wolves and silver foxes [26]. The lack of seasonality in most breeds of domestic dogs might indicate that the oestrus cycle is independent of the light cycle and therefore possibly of melatonin.

There seems to be a lack of knowledge of normal ranges in serum melatonin concentrations in the dog. In addition, there are very large variations in ranges between different studies. Ferreira et al. (2020), using an ELISA, reported values of 18.5–244.9 pg/mL [26,27] in 38 dogs. Ashley et al. (1999), also using an ELISA, reported somewhat similar values with mean baseline concentrations of 137 pg/mL and all values exceeding the upper detection limit of 300 pg/mL up to eight hours after oral administration of 25 mg of melatonin. Attempts to access serum concentrations >300 pg/mL by serial dilutions were unsuccessful in 3 of 4 dogs. The remaining dog had serum concentrations of 311–624 pg/mL 30–240 min after oral administration of melatonin, similar to values in the present study [21]. In contrast, some studies report much lower serum concentrations of melatonin in the dog. Zan et al. (2013), using an ELISA kit, reported mean values below 14 pg/mL, varying with time of day and season in a pilot study with 6 dogs [28]. Dunlap et al. (2007), using a radioimmunoassay, reported similar results with mean values <20 pg/mL, also with diurnal and seasonal changes [20]. Thomovsky et al. (2019) reported even lower values, using a radioimmunoassay, with a majority of dogs having values below the lowest detection limit of 0.5 pg/mL and the highest value being 1.84 pg/mL. They also reported values of >5000 pg/mL in four pilot dogs supplemented with oral melatonin [29]. Thomovsky et al. (2019) found no effect of season [29], similar to the results of our study. In many studies on melatonin in the domestic dog or cat, serum concentrations of melatonin are not mentioned or evaluated [7,12,29,30,31,32]. Based on the lack of knowledge about expected serum melatonin values in the domestic dog, it is difficult to relate the results of this study to previous reports.

There was no consistent increase in serum melatonin concentrations after treatment in this study. It is possible that the large variation in serum melatonin concentrations might have obscured a treatment effect. It is unlikely that time of sampling affected the results, as all samples were collected during daytime (between 8:00 and 17:00) [20,28]. Time after treatment might have affected the results. However, according to the product sheet, the implants release melatonin progressively in sufficient concentrations in the sheep for 70–90 days; therefore, it would be reasonable to expect an effect within a month after implantation in the dog [10]. Dunlap et al. [20] reported that the factor that showed the most pronounced reduction in plasma melatonin in sled dogs was exercise. Therefore, the possibility that spontaneous activity levels in the bitches in the current study may have affected serum melatonin concentrations cannot be excluded. It is also possible that a larger study group would have been necessary to detect an increase, considering the large variations in serum concentrations. Similar to our study, Gianetto et al. [33] found no effect of oral melatonin treatment on serum melatonin concentrations despite an effect on tear melatonin concentrations. In the sheep, it was reported that there was a breed effect on serum melatonin concentrations after implantation [34]. 

No side effects were observed in this study, similar to the study of Verschuren et al. [32], who used the same type of implants as in the current study. We did not even observe swelling at the injection site, as was observed in 3/8 dogs in their study. 

There were some limitations in the study. Because of the long oestrus cycle in the bitch and the necessity to follow bitches for three cycles, only research colony dogs could be used. This limited the number of animals that were available. It has been stated that the pharmakokinetics and therapeutic doses of melatonin in dogs are poorly understood [31], and there seems to be a lack of knowledge about normal basal serum concentrations of melatonin in the domestic dog, with very large variations between studies.

Similar to the results in this study, 2.0 mg of oral melatonin daily did not affect reproductive events in the grey wolf [24]. It is still not known if melatonin is involved in the control of cyclicity in the domestic dog. The dose of melatonin might, however, be an important factor. Although the dose used in this study was efficient in altering cyclicity in the sheep and the domestic cat, the domestic dog may be less sensitive and require higher doses for an effect. Daily oral melatonin might be more efficient, although less practical, and was not efficient in altering reproductive events in the grey wolf. 

## 5. Conclusions

The results demonstrated that implants of 18 mg of melatonin are not likely to be a useful method to control the oestrus cycle in the domestic dog, in contrast to the sheep and the domestic cat.

## Figures and Tables

**Table 1:** Interoestrus intervals and time to pro-oestrus after treatment in individual bitches.

Bitch	Group	First Interoestrus Interval (Untreated) (Days)	Second Interoestrus Interval (after Treatment) (Days)	Difference in Interval Length (Days)	Treatment/Control to Oestrus
1	Control	189	203	14	47
2	Control	199	211	12	42
3	Control	210	206	−4	71
4	Control	267	209	−58	0 *
5	Implant	160	168	8	40
6	Implant	241	232	−9	18
7	Implant	240	235	−5	28
8	Implant	205	201	−4	8
9	Implant	240	235	−5	28

* Heat had started at the time of sham implant, which was before the planned time for entering the control group.

**Table 2:** Serum melatonin concentrations (pg/mL) in individual bitches in relation to treatment.

Bitch	Group	Melatonin at Implant or Entering Control Group	Melatonin Post-Treatment or Control	Interval between Samples
1	Control	105.3	74.8	11
2	Control	309.6	517.9	11
3	Control	1363.3	1592.5	30
4	Control	141.6 *	391.0	8
5	Implant	636.3	506.4	22
6	Implant	130.9	138.7 ^#^	18
7	Implant	412.7	391.1	14
8	Implant	118.8	314.1 ^#^	13
9	Implant	229.5	234.4	14

* Pro-oestrus started before the planned time for entering the control group; blood sample was therefore collected at the start of pro-oestrus. ^#^ Pro-oestrus at the time of the post-implant sample.

## Data Availability

Datasets are available from the author on reasonable request.
